# Thematic Selection: The Comorbidity of Mental Disorders and Metabolic Disorders (Part I)

**DOI:** 10.2174/1570159X2307250221124221

**Published:** 2025-02-21

**Authors:** Zezhi Li

**Affiliations:** 1 Department of Nutritional and Metabolic Psychiatry, Affiliated Brain Hospital of Guangzhou Medical University, Guangzhou, China;; 2 Guangdong Engineering Technology Research Center for Translational Medicine of Mental Disorders, Guangzhou, China;; 3 Key Laboratory of Neurogenetics and Channelopathies of Guangdong Province and the Ministry of Education of China, Guangzhou Medical University, Guangzhou, China;

The comorbidity of mental disorders and metabolic disorders refers to the frequent occurrence of metabolic abnormalities in patients with mental disorders, and conversely, patients with metabolic diseases may also experience mental health problems. This comorbidity is very common in clinical practice and has a significant impact on the overall health and treatment outcomes of patients. Patients with mental disorders are more likely to experience metabolic dysregulation due to factors such as medication treatment, lifestyle, and psychological status [1-4]. Conversely, metabolic abnormalities can worsen the symptoms of mental disorders, increasing the difficulty of treatment [5]. Therefore, the comorbidity of mental disorders and metabolic disorders not only affects the physical health of patient but also has profound implications for their mental state, treatment efficacy, and quality of life.

In recent years, research on the comorbidity of mental disorders and metabolic disorders has attracted increasing attention from scholars, and rapid progress has been made in this field. Numerous studies have revealed the biological mechanisms underlying the relationship between mental disorders and metabolic disorders, highlighting the complex and multidimensional nature of their connection. The causes of comorbidity involve multiple factors, including inflammatory responses in the body, neuroendocrine dysregulation, imbalance of the gut-brain axis, interactions between neurons and adipocytes, the impact of psychiatric medications on metabolism, as well as changes in appetite and eating behaviors [3, 6-8].

The comorbidity of mental disorders and metabolic disorders presents significant challenges for treatment. First, the pharmacological treatment of mental disorders, especially antipsychotic medications, is often associated with metabolic side effects such as weight gain, impaired glucose tolerance, and hyperlipidemia, which can exacerbate metabolic issues in patients. Second, the lifestyle factors of the patient, such as irregular eating habits, lack of exercise, and sleep disturbances, further contribute to the development of metabolic abnormalities. Therefore, treating these patients requires a comprehensive approach that takes into account the side effects of medications, lifestyle interventions, and psychological treatments, adopting a personalized and multi-faceted treatment strategy (*e.g*., primary care intervention) [9]. At the same time, emerging therapeutic approaches are being explored, such as gut microbiome interventions, which are seen as a potential strategy to simultaneously address symptoms of both metabolic diseases and mental disorders [10-12]. Modulating the gut microbiome may improve brain function by altering neuroendocrine and immune responses [13], thereby alleviating mental symptoms and helping restore normal metabolic function.

Future research will also explore the metabolic effects of different types of drugs (such as GLP-1 receptor agonists, SGLT2 inhibitors, *etc*.) on patients with mental disorders like depression and anxiety while evaluating the dual effects of these drugs on mood and metabolism. GLP-1 receptor agonists have shown significant efficacy in diabetes treatment, and studies suggest they may have the potential to improve mental disorders as well [14, 15]. SGLT2 inhibitors, primarily used for treating type 2 diabetes, have also garnered attention for their effects on cardiovascular health and weight reduction [16]. By assessing the dual impact of these medications on mood and metabolism, new solutions may emerge for the treatment of comorbid mental disorders and metabolic disorders.

This editorial introduces a series of articles that explore the latest research on the biological mechanisms, clinical features, risk factors, and treatment approaches for mental disorders and metabolic disorder comorbidity. The articles cover topics ranging from the neurobiological underpinnings of comorbidity to innovative interventions aimed at improving both metabolic and mental health outcomes. Our aim is to foster a deeper understanding of how these two realms interact and to encourage the development of integrated therapeutic strategies that can more effectively address the complex needs of these patients.

As we continue to unravel the complexities of mental and metabolic health, it is clear that a holistic, interdisciplinary approach is essential. This issue aims to provide valuable insights that will shape future research and clinical practices, ultimately improving the care and outcomes for patients affected by both mental and metabolic disorders.

## FUNDING

This study was funded by the Natural Science Foundation of Guangdong Province (2023A1515011728), Guangzhou Municiple Health Commission (2023C-TS26), Plan on enhancing scientific research in GMU (02-410-230221XM), Project of Guangzhou Municipal Science and Technology Bureau (2023A03J0835), Guangzhou Municipal Key Discipline in Medicine (2025-2027), Guangzhou High-level Clinical Key Specialty, and Guangzhou Research-oriented Hospital. All funding had no role in study design, data analysis, paper submission and publication.
